# Functional flexible adsorbents and their potential utility

**DOI:** 10.1039/d4cc05393a

**Published:** 2025-01-20

**Authors:** Kyriaki Koupepidou, Aizhamal Subanbekova, Michael J. Zaworotko

**Affiliations:** a Bernal Institute, Department of Chemical Sciences, University of Limerick Limerick V94T9PX Republic of Ireland xtal@ul.ie

## Abstract

Physisorbents are poised to address global challenges such as CO_2_ capture, mitigation of water scarcity and energy-efficient commodity gas storage and separation. Rigid physisorbents, *i.e.* those adsorbents that retain their structures upon gas or vapour exposure, are well studied in this context. Conversely, cooperatively flexible physisorbents undergo long-range structural transformations stimulated by guest exposure. Discovered serendipitously, flexible adsorbents have generally been regarded as scientific curiosities, which has contributed to misconceptions about their potential utility. Recently, increased scientific interest and insight into the properties of flexible adsorbents has afforded materials whose performance suggests that flexible adsorbents can compete with rigid adsorbents for both storage and separation applications. With respect to gas storage, adsorbents that undergo guest-induced phase transformations between low and high porosity phases in the right pressure range can offer improved working capacity and heat management, as exemplified by studies on adsorbed natural gas storage. For gas and vapour separations, the very nature of flexible adsorbents means that they can undergo induced fit mechanisms of guest binding, *i.e.* the adsorbent can adapt to a specific adsorbate. Such flexible adsorbents have set several new benchmarks for certain hydrocarbon separations in terms of selectivity and separation performance. This Feature Article reviews progress made by us and others towards the crystal engineering (design and control) of flexible adsorbents and addresses several of the myths that have emerged since their initial discovery, particularly with respect to those performance parameters of relevance to natural gas storage, water harvesting and hydrocarbon gas/vapour separation.

## Abstract 2

Flexible porous materials that exhibit extreme guest-induced phase transformations are under valued in terms of their potential utility. As detailed herein, recent advances in understanding and properties of such adsorbents should change this view.

## Graphical abstract

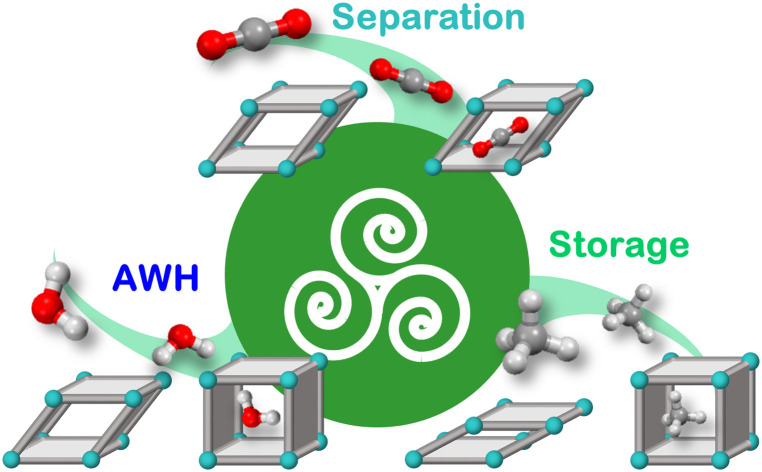

## Introduction

The International Union of Pure and Applied Chemistry (IUPAC) defines an adsorbent as a condensed phase at the surface of which adsorption may occur.^1^ Adsorbents serve important everyday applications in separation and purification such as gas separation and recovery,^2,3^ water decontamination^4,5^ and air purification,^6,7^ and they are of topical interest for water harvesting^8^ and gas storage applications.^9,10^ Adsorbents can be conveniently classified by three criteria that influence their functional properties, (i) mechanism of adsorption, (ii) degree of porosity and (iii) composition:

(i) Physisorption or chemisorption. Physisorbents rely on physical adsorption (physisorption),^11^*i.e.* adsorbent–adsorbate interactions result from weak noncovalent forces. Chemisorbents exploit covalent bond breakage and formation (chemisorption).^12^

(ii) Nonporous or porous. Porous adsorbents such as zeolites,^13^ most metal–organic frameworks (MOFs)^14^ and some hydrogen bonded organic frameworks (HOFs)^15^ or halogen bonded organic frameworks (XOFs)^16^ exhibit internal porosity,^17^ whereas nonporous adsorbents such as silica and carbon black do not.

(iii) Network or molecular solid. Network solids such as diamond and MOFs are sustained by covalent bonds. Molecular solids such as organic inclusion compounds^18^ are sustained by noncovalent forces.^19^

Physisorbents are of topical interest for their potential utility since they generally require less energy for desorption of the adsorbate than chemisorbents.^20^ Porous adsorbents tend to be preferred as they can have an internal surface area in addition to external surface area, meaning that their uptakes are typically higher than nonporous adsorbents.^21^ There should be no inherent prejudice as to whether an adsorbent is a molecular or network solid, but their nature can profoundly impact mechanism and performance.

**Fig. 1:**
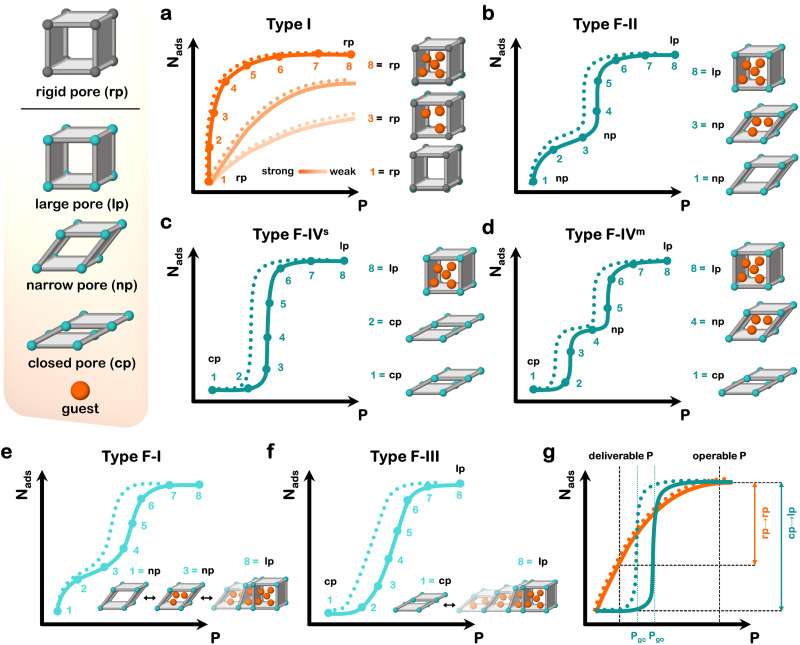
Schematic representations of the rigid pore (rp) structure of a rigid adsorbent and of the large pore (lp), narrow pore (np), closed pore (cp) structures of a flexible adsorbent and guest, corresponding to (a) type I isotherm, or sudden phase transformations resulting in (b) type F-II, (c) type F-IV^s^ (s = single-step) and (d) type F-IV^m^ (m = multistep) isotherms. Gradual phase transformations result in (e) type F-I and (f) type F-III isotherms. (g) Comparison of working capacity between deliverable pressure (deliverable *P*) and operable pressure (operable *P*) for type I (orange) and F-IV^s^ (cyan) isotherms with similar surface areas. Gate-opening (*P*_go_) and gate-closing (*P*_gc_) pressures in (g) are shown for a flexible adsorbent. Adsorption = solid line; desorption = dotted line. Different shades in (a) indicate different strength of adsorbent–adsorbate interactions: dark to light orange = strong to weak.

Most porous physisorbents are considered to be “rigid” adsorbents, *i.e.* they retain their structures during gas or vapour sorption cycles. Both molecular and network solids that behave as rigid adsorbents were studied decades ago by Barrer, as exemplified by Dianin's compound^22^ and zeolites.^23^ Zeolites can be naturally occurring^24^ or synthetic,^25^ with 256 zeolite framework topologies archived in the International Zeolite Association (IZA) database.^26^ MOFs and coordination networks (CNs)^27–29^ such as hybrid ultramicroporous materials (HUMs)^30^ have emerged in the past three decades and differ from zeolites as they are highly modular in terms of their chemical composition. This makes them amenable to design using principles such as crystal engineering,^31–33^ so that a particular topology can have hundreds or even thousands of related or isoreticular^34^ members of the same family. The first reports of permanent porosity in MOFs were made in 1997 by the groups of Kitagawa and Mori, Co_2_(4,4′-bpy)_3_(NO_3_)_4_,^35^ and Cu(ii) terephthalate, Cu(bdc),^36^ respectively, and in 1998 by Yaghi's group, Zn(bdc).^37^ The gas sorption isotherms of these adsorbents are concave to the pressure axis, which is classified by IUPAC as a type I isotherm^38^ and corresponds to loading of a rigid pore (rp, Fig. 1a). The slope of the curve is related to the strength and uniformity of adsorbent–adsorbate interactions. Many high surface area MOFs exhibit weak adsorbent–adsorbate interactions resulting in nearly linear adsorption profiles, whereas HUMs can offer steeper uptakes at low pressure due to strong adsorbent–adsorbate interactions (Fig. 1a).^39^

Flexible adsorbents, *i.e.* adsorbents that undergo structural transformation(s) upon guest exposure, were to our knowledge first reported by Barrer for a Werner complex, a nonporous molecular solid, in 1969.^40^ Although the crystal structures corresponding to the guest-induced phase transformations were not then reported, this work laid the groundwork for what would later be called flexible adsorbents. Prediction of flexible adsorbents in the then emerging field of MOFs was made in 1998 by Kitagawa,^41,42^ validated three years later when ELM-11(Cu) was reported by Kaneko.^43^ It was noted that the observed isotherm types, which typically involve one or more steps, “*cannot be classified into the representative six types recommended by IUPAC*”.^43^ Today, a relatively small but growing subset of MOFs (perhaps ≤ 1%) are known to exhibit flexibility.^44,45^ We herein define a flexible adsorbent as an adsorbent that undergoes a guest-induced structural transformation during gas, vapour or liquid sorption, resulting in changes to its pore size, shape and/or chemistry, as usually evidenced by *in situ* structural characterisation. Structural characterisation is typically necessary to validate that phase transformations have indeed occurred during gas or vapour uptake. Whereas a stepped adsorption isotherm can be an indicator of flexibility, it is not definitive, as stepped isotherms can also occur in rigid adsorbents for vapours and liquids.^46^ Although flexibility can manifest in both global and local dynamics,^47^ locally dynamic adsorbents exhibit only localised structural changes without long-range transformations in response to guest exposure. In contrast, globally dynamic or “cooperatively flexible” adsorbents, herein referred to as flexible adsorbents, undergo discontinuous phase changes leading to stepped isotherms, and are the focus of this Feature Article.

Flexible adsorbents tend to exhibit distinctive isotherm “steps” (Fig. 1b–f).^48^ Although some of the isotherm types defined by IUPAC are similar in shape, they are associated with monolayer or multilayer adsorption in which the surface layer of the adsorbent is equally accessible to the adsorbate.^49^ However, this is not the case in flexible adsorbents as the initial phase has less (or no) accessible surface, whereas the subsequent guest-loaded phase does. Therefore, additional isotherm types must be defined to reflect sorption in flexible adsorbents. Isotherm step(s) are associated with structural transformation(s), typically (but not always) from a less porous (narrow pore; **np**) or nonporous (closed pore; cp) activated phase to a more porous (large pore; lp) loaded phase. The nature of the initial phase impacts the profile of the isotherm before the step: np phases result in type F-I (gradual) or F-II (sudden) isotherms (np → lp; Fig. 1b and e); cp phases afford type F-III (gradual) or F-IV (sudden) isotherms (cp → lp; Fig. 1c and f). Sharp isotherm steps corresponding to cp → lp transformations can be classified as “switching”,^50^ and can involve one (cp → lp; type F-IV^s^, where “s” stands for single-step isotherm; Fig. 1c) or multiple (cp → np → lp; type F-IV^m^, where “m” stands for multistep isotherm; Fig. 1d) steps depending on the adsorbent and adsorbate. Hysteresis, where the adsorption and desorption curves do not match is typically a result of kinetic barriers and metastable phases.^41,51^ The pressure threshold at which a step occurs during adsorption and desorption can be termed the gate-opening (*P*_go_) and gate-closing (*P*_gc_) pressures, respectively.

Insight into the underlying mechanisms behind different isotherm profiles is typically gained through *in situ* structural characterisation, with computational modelling being a powerful complementary technique to provide further insight. The increasing availability and utilisation of *in situ* X-ray diffraction (XRD) and advances in computational modelling have provided insight into the design and properties of flexible adsorbents and enable us to better assess their potential utility. The sorption behaviour of flexible adsorbents suggests possible advantages: (i) enhanced working capacity; (ii) intrinsic thermal management; (iii) improved molecular recognition and selectivity. Working capacity, *i.e.* the uptake difference between deliverable (high) and operable (low) pressures, can be higher in flexible adsorbents than rigid ones. This is because rigid adsorbents tend to retain gas at the deliverable pressure, therefore limiting the uptake difference between deliverable and operable pressures (Fig. 1g). A flexible adsorbent with a type F-IV^s^ isotherm (Fig. 1c) retains no gas at deliverable pressure, if *P*_gc_ is above the deliverable pressure. That flexible adsorbents undergo an endothermic phase transformation induced by gas pressure can partly offset exothermic heat release during adsorption. Finally, as phase transformations are guest-induced, flexible adsorbents can exhibit molecular recognition (selectivity) if a phase transformation is induced by only one component of a mixture.

With increasing scientific studies concerning flexible adsorbents, several misconceptions or “myths” have emerged, all of which seem intuitive but would mitigate against utility: lack of design principles to identify parent (first generation, Gen 1) adsorbents; hydrolytic or mechanical instability, a feature of many MOFs; slow kinetics *vs.* rigid adsorbents since structural transformation(s) are required; hysteresis; low gas uptake because phase changes are not extreme enough; lack of selectivity resulting from co-adsorption in lp phases. Although many reviews have previously highlighted the potential utility of flexible adsorbents,^47,52–55^ none of them have directly addressed these misconceptions. This Feature Article provides the state-of-the-art in the context of these perceived weaknesses by addressing design and control principles from a crystal engineering perspective, before addressing each misconception through examples of flexible adsorbents studied in our group. We intend for this review to emphasise that functional flexible adsorbents are now candidates for utility in natural gas storage, water harvesting and hydrocarbon gas/vapour separation.

## Chronology of key discoveries in flexible adsorbents for energy applications

**Fig. 2:**
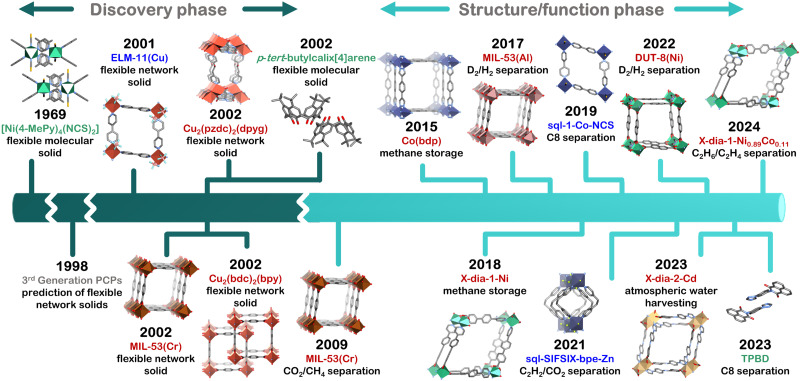
Chronology of the “discovery” (dark cyan) and “structure/function” (light cyan) phases of the development of flexible adsorbents, for which representative isotherms corresponding to phase transformations were reported. Compound names are colour coded for molecular (green) solids, 2D network (blue) and 3D network (red) solids. Crystal structures are generated from .cif files archived in the Cambridge Structural Database (ref. 45). CCDC numbers: 1179557 ([Ni(4-MePy)_4_(NCS)_2_]), 974376 (ELM-11(Cu)), 797261 (MIL-53(Cr)), 863312 (Cu_2_(bdc)_2_(bpy)), 168595 (Cu_2_(pzdc)_2_(dpyg)), 1242903 (*p-tert*-butylcalix[4]arene), 1058444 (Co(bdp)), 1818655 (sql-1-Co-NCS), 2048415 (sql-SIFSIX-bpe-Zn), 2020029 (DUT-8(Ni)) 2240524 (X-dia-2-Cd), 2243659 (TPBD), 1426847 (X-dia-1-Ni). Atom colours: C, grey; N, blue; O, red; S, yellow; F, neon green; B, pink; Cu, orange; Cr, burnt orange; Co, indigo; Ni, green; Zn, purple; Cd, gold; Al, dusty pink. Hydrogen atoms are omitted for clarity.

Since Barrer introduced the first flexible adsorbent, [Ni(4-MePy)_4_(NCS)_2_],^40^ both network and molecular solids have been studied (Fig. 2). Kitagawa's prediction of “third-generation” flexible porous coordination polymers, PCPs,^42^ was validated by Kaneko *et al.* when they reported a two-dimensional (2D) framework, Cu(bpy)_2_(BF_4_)_2_, in 2001 (now commonly known as ELM-11(Cu)).^43^ Three-dimensional (3D) flexible MOFs were reported in the following year, including Cu_2_(bdc)_2_(bpy) by Seki,^56,57^Cu_2_(pzdc)_2_(dpyg) by Kitagawa,^58^ and MIL-53(Cr) by Férey and Serre.^59^ Also in 2002, flexibility was reported for the nonporous organic molecular solid *p-tert*-butylcalix[4]arene by Atwood and Barbour.^60^ However, practical applications of flexible adsorbents remained understudied until 2009, when MIL-53(Cr) was investigated for CO_2_/CH_4_ separation.^61^ Long's report concerning Co(bdp) for CH_4_ storage in 2015 enabled flexible adsorbents to gain momentum for energy applications.^62^ In 2017, MIL-53(Al) demonstrated the separation of hydrogen isotopes, with selectivity for D_2_ over H_2_.^63^ In 2018, our group reported high working capacity for CH_4_ storage by X-dia-1-Ni^48^ and one year later selective C8 separation was reported for sql-1-Co-NCS.^64^ Our study of induced fit binding of C_2_H_2_ by sql-SIFSIX-bpe-Zn in 2021 set a new benchmark for C_2_H_2_ binding and C_2_H_2_/CO_2_ selectivity.^65^ The extensively studied DUT-8(Ni) introduced by Kaskel's group was found to exhibit D_2_/H_2_ separation in 2022 through selective cp → lp transformation for D_2_.^66^X-dia-2-Cd was found in 2023 to be the first flexible adsorbent meeting criteria for atmospheric water harvesting,^67^ while a molecular solid, TPBD, exhibited benchmark *p*-xylene binding.^68^ Most recently, cobalt doping of X-dia-1-Ni afforded X-dia-1-Ni_0.89_Co_0.11_, which exhibited inverse C_2_H_6_/C_2_H_4_ selectivity.^69^ Interestingly, these reports do not fully address composition/property relationships. The next section therefore addresses if flexibility can be expected:

Myth 1: ***“Flexible adsorbents are curiosities that are difficult to design or control.”***

Once a Gen 1 adsorbent has been identified, the inherent modularity of CNs and most molecular compounds allows for crystal engineering of families of second generation (Gen 2) flexible adsorbents through systematic substitution of components. CNs based on molecular building blocks (MBBs)^31^ can provide platforms based on the most commonly observed and accessible topologies:^70,71^ square lattice (sql), primitive cubic (pcu) and diamondoid (dia). Indeed, sql-1-Co-NCS belongs to the same sql family as ELM-11(Cu), X-dia-2-Cd has dia topology like X-dia-1-Ni, and DUT-8(Ni) has pcu topology as does Cu_2_(bdc)_2_(bpy). Network solids based on rod building blocks (RBBs)^72^ include MIL-53(Cr) and Co(bdp). The primary issue is, therefore, not systematic development of Gen 2 materials, but rather discovery of the prototypal Gen 1 member. In this context, mechanisms of flexibility can provide guidance.

## Design and control of flexible adsorbents

Flexible adsorbents undergo structural changes upon both guest removal and introduction. Upon guest removal, this flexibility originates from transitions to more thermodynamically favourable states *via* flexibility mechanisms, which promote densification and frequently facilitate the formation of exothermic noncovalent interactions within the adsorbent.^47^ The subsequent flexibility exhibited upon guest introduction is facilitated through compensatory host–guest interactions. Understanding these mechanisms of flexibility is a prerequisite for control and fine-tuning of flexible adsorbents. While CNs and Werner complexes can be readily fine-tuned by metal and (equatorial and axial) ligand substitution, organic molecular solids can only be tuned by covalent bond modification. The high degree of modularity of CNs and Werner complexes affords several variables from a crystal engineering perspective,^73^ making them the primary focus of this section.

### Flexibility in molecular solids

**Fig. 3:**
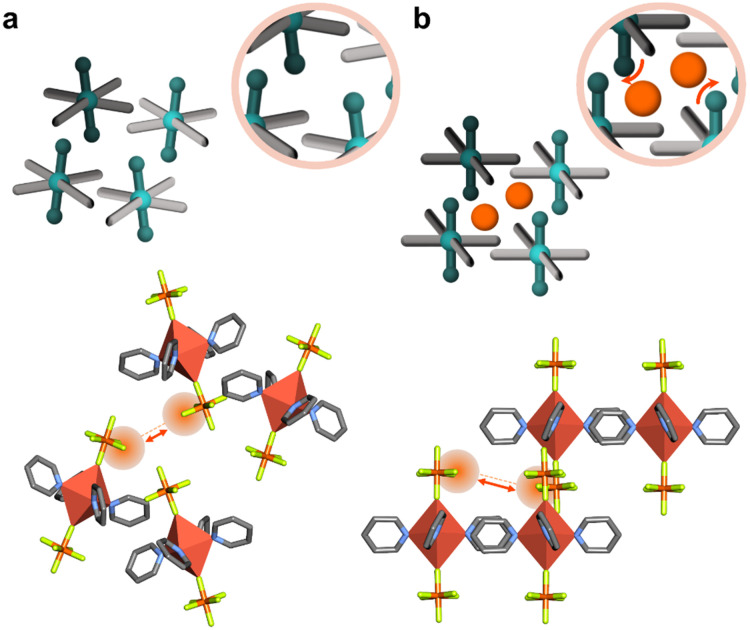
Phase transformation in Werner complex [Cu(Py)_4_(PF_6_)_2_], (a) guest-free and (b) guest-loaded structure. Crystal structures generated from .cif files archived in the Cambridge Structural Database. CCDC numbers: 870626, 870630. Atom colours: C, grey; N, blue; F, neon green; Cu, dark orange; P, light orange. Hydrogen atoms are omitted for clarity.

In molecular solids, crystal packing can change to accommodate guest molecules within pockets formed by adjacent molecular units. Early examples of organic and metal-organic molecular solids where both the guest-loaded and guest-free phases were characterized are *p-tert*-butylcalix[4]arene and [Ni(4-MePy)_4_(NCS)_2_].^40,60,74^ Both exist as nonporous guest-free solids that undergo structural transformation through rearrangement of noncovalent bonds: for *p-tert*-butylcalix[4]arene, ab/cd packing in the cp phase changes to ab/ab packing to accommodate two aromatic molecules; for [Ni(4-MePy)_4_(NCS)_2_], translation between adjacent complexes creates a cavity to fit one benzene molecule. Werner complexes can be readily modified through ligand or metal substitution. For example, Barrer also reported gas sorption for the Co analogue of [Ni(4-MePy)_4_(NCS)_2_]. Further, [Co(4-EtPy)_4_(NCS)_2_] was found to exhibit phase transformations when exposed to benzene, toluene and C8 isomers (where 4-MePy = 4-methylpyridine and 4-EtPy = 4-ethylpyridine).^40^ Pyridine (Py) ligands were investigated in the series [Cu(Py)_4_X_2_] (X = PF_6_, BF_4_, CF_3_SO_3_, CH_3_SO_3_), with [Cu(Py)_4_(PF_6_)_2_] and [Cu(Py)_4_(BF_4_)_2_] exhibiting inflected isotherms for acetonitrile (298 K) and methanol (283 K).^75^ The phase transformations for [Cu(Py)_4_(PF_6_)_2_] are consistent with Barrer's earlier studies (Fig. 3).

In order to find more examples of molecular solids that can act as adsorbents, our approach is to first identify host–guest complexes in “as-synthesised” crystal structures, including hydrates and solvates. Werner complexes have been known to form Werner clathrates since the late 1950s,^76^ and several have since been found to exhibit reversible guest-induced structural transformations. Identification of host–guest complexes can aid the design of flexible adsorbents by studying what happens after the removal of guests. For example, we recently revisited the Werner complex [Ni(4-MePy)_4_(NCS)_2_], which was reported to form clathrates with aromatic molecules in 1957,^77^ while its nonporous phase was reported in 1952.^78^ By identifying these phases we found that [Ni(4-MePy)_4_(NCS)_2_] can serve as a flexible adsorbent for *p*-xylene and that it shows a rarely observed shape-memory effect.^79^ Solvate or hydrate formation is therefore one indicator of a potential flexible adsorbent. Organic molecular solids that are promiscuous with respect to conformational polymorphism is another indicator, an example of which is discussed in the separation section.^68^

### Flexibility in network solids

#### Ligand types that enable flexibility

**Fig. 4:**
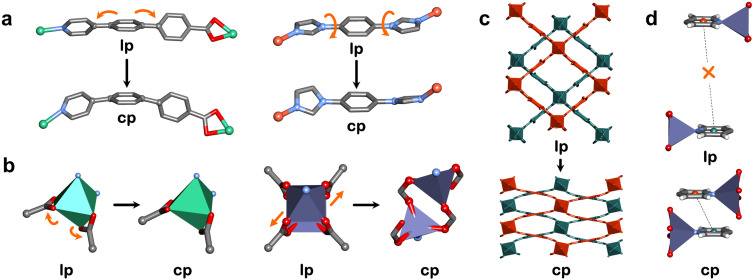
(a) Ligand changes in X-dia-1-Ni (left) and SIFSIX-23-Cu (right), (b) MBB changes in X-dia-1-Ni (left) and X-pcu-5-Zn (right) and (c) interpenetration changes (*i.e.* subnetwork displacement) in X-pcu-5-Zn during large pore (lp) to closed pore (cp) phase transformations. (d) Noncovalent interaction (displaced π–π interaction) between two individual networks in the cp phase of X-pcu-5-Zn and absence of the same interaction in the lp phase. Crystal structures generated from .cif files archived in the Cambridge Structural Database. CCDC numbers: 1426847, 1426850, 1961593, 1961595, 1856628, 1856629, 1856631. Atom colours: C, grey; N, blue; O, red; Cu, orange; Ni, green; Zn, purple. Hydrogen atoms are omitted for clarity.

Linker ligands are key to rational design of CNs and the choice of a ligand that is inherently flexible might be expected to afford flexible CNs. In our group, we initially targeted extended ligands, “X ligands”, that are able to contort and change the orientation of MBBs. We reported our first example in 2018, X-dia-1-Ni (Fig. 4a).^48^ The ligand in X-dia-1-Ni (1 = 4-(4-pyridyl)-biphenyl-4-carboxylic acid) contorted from nearly planar (174°) in the lp phase to bent (155°) in the cp phase (determined by the angle between the N atom of the pyridyl ring and the C atom of the carboxylate group). The X ligand approach is applicable to other platforms and typically exploits ligands with three or more consecutive aromatic rings arranged in linear fashion, such as in X-dia-2-Cd and X-sql-1-Cu (2 and 1 = 4-((4-(1*H*-imidazol-1-yl)phenylimino)methyl)benzoic acid).^67,80^ Soon after, we found that ligands that are able to undergo *cis*/*trans* or *syn*/*anti* isomerisation, can also enable dramatic structural transformations. Specifically, in SIFSIX-23-Cu (23 = 1,4-bis(1*H*-imidazol-1-yl)benzene), the linker ligand isomerised from *syn* to *anti* conformations with shorter M–M distances in the *syn* conformation (Fig. 4a).^81^ Linear bisimidazole ligands have also been used in several other platforms^82–84^ and similar structural changes were observed for bent bisimidazole ligands.^85^ More recently, we explored the incorporation of pendant groups on linkers to afford turnstile motions that instigate phase transformations in X-dmp-1-Co (1 = 2,5-diphenylbenzene-1,4-dicarboxylate). The peripheral phenyl groups on the carboxylate ligand were responsible for a turnstile effect that enabled guest-induced cp → cp phase transformations (transient porosity).^86^ Additionally, the use of bulky pendant groups (R = –CH_3_, –OCH_3_, –C(CH_3_)_3_, –N=N–Ph, and –N=N–Ph(CH_3_)_2_) resulted in a new platform of flexible adsorbents with sql topology, Zn(5-Ria)(bphy) (ia = isophthalic acid and bphy = 1,2-bis(pyridin-4-yl)hydrazine).^87^

Based on the above, we can categorise our research efforts into three categories of linker ligands that offer potential for flexibility: (i) linkers that contort (X ligands); (ii) linkers that isomerise (*e.g. syn*/*anti*); (iii) linkers with pendant groups. However, these results do not preclude the use of rigid ligands in flexible adsorbents, as there are other features that can promote flexibility. Indeed, rigid ligands such as 4,4′-bipyridine, which propagate linear linkages between coordination sites, cannot induce flexibility on their own. Nevertheless, they are frequently encountered in flexible adsorbents as there are other mechanisms that can induce flexibility.

#### Metal node types that enable flexibility

Metal-based nodes can be inherently capable of inducing flexibility even where the linker ligand is rigid. Though they are not isolated nodes (MBBs), metal-based RBBs, which are in effect infinite nodes, can enable a variety of coordination angles that support phase transformations. As such, the prototypal flexible CNs MIL-53(Cr) and MIL-47(V) rely on carboxylate RBBs, while Co(bdp) is comprised of a pyrazolate RBB. Both RBBs enable hinge-like motions.^59,62,88,89^

Our approach has focused on MBBs that distort or isomerise. The simplest MBBs comprise a single metal moiety where O–M–O, N–M–O and N–M–N angles can vary (O = oxygen, N = nitrogen, M = metal). This was observed in ZIF-7 (ZIF = zeolitic imidazolate framework), where flexibility primarily originates from the flapping motion of the rigid benzimidazolate linker ligand around the tetrahedral MBBs.^90^ In our studies, octahedral distortion was seen in X-dia-1-Ni (Fig. 4b).^48^ In other cases, breakage of one of the chelating M–O bonds of a carboxylate moiety can result in change of geometry, *e.g.* octahedral to tetrahedral, as seen in X-dia-4-Co and X-dia-5-Co.^83^ Whereas bond angle deformations in monometallic MBBs are typically subtle, metal cluster MBBs can undergo more extreme deformations. For example, isomerisation of the paddle-wheel MBB in X-pcu-5-Zn by changing the coordination mode of the O-donor linker from bidentate to monodentate resulted in phase transformation to a new cp polymorph (Fig. 4b).^91^ This isomerisation resulted in the geometry changing from square pyramidal to tetrahedral, which is more expected for Zn than Co, Ni or Cu due its d^10^ electronic configuration. Such a situation is exemplified by DUT-8(Ni) and DUT-8(Co), which retained square pyramidal geometry after phase transformation, whereas DUT-8(Zn) underwent isomerisation.^92,93^ More recently, we reported a new MBB that enables deformations similar to RBBs by mimicking a butterfly waving its wings (“butterfly” motions). We employed this MBB in a double-walled dia network (X-dia-6-Ni)^94^ as well as a double diamondoid (ddi) network (X-ddi-1-Ni).^82^

#### Other mechanisms of flexibility

**Fig. 5:**
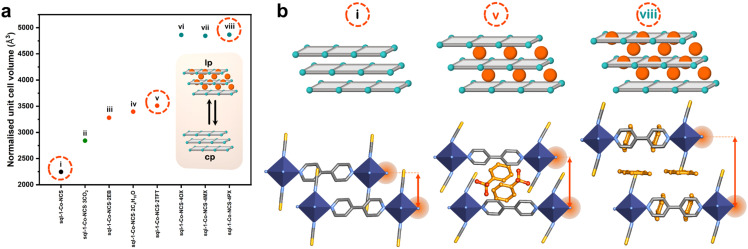
(a) Unit cell volume (normalised for *Z* = 4) corresponding to different phases of **sql-1-Co-NCS** (i)–(viii). (b) Schematic representations of layer packing in **sql-1-Co-NCS** (i), **sql-1-Co-NCS·2TFT** ((v); TFT = α,α,α-trifluorotoluene) and **sql-1-Co-NCS·4PX** ((viii); PX = *p*-xylene). Crystal structures generated from .cif files archived in the Cambridge Structural Database. CCDC numbers: 767579, 1818653 and 1818657. Atom colours: C, grey; N, blue; S, yellow; Co, indigo. Guest molecules are shown in ball and stick representations (C, light orange; F, dark orange). Hydrogen atoms are omitted for clarity.

Layer expansion (in 2D CNs) and network displacement (in interpenetrated 3D CNs) can also drive flexibility. Whereas phase transformations can involve more than one mechanism, one mechanism might dominate in a particular class of CNs. For instance, sql networks can accommodate guest molecules in their interlayer spaces and/or within intranetwork cavities. As such, their layer structure can remain mainly unaltered with interlayer spaces expanding or contracting in clay-like fashion (Fig. 5). Layer expansion tends to occur through breakage of internetwork noncovalent bonds between layers upon guest inclusion. Such a mechanism was proposed by Kaneko, who hypothesised that the hydrogen bonds between 2D layers of ELM-11(Cu) break upon gas loading, allowing the layers to rearrange to accommodate gas molecules in a 1D channel.^43^ This is indeed the situation for other switching layered adsorbent materials (SALMAs).^64,95^ Recently, in collaboration with Xu and Kaskel, we demonstrated that the size and number of adsorbate molecules per formula unit can be correlated to the degree of layer expansion in sql-1-Ni-NCS,^95^ with larger or higher number of adsorbates inducing longer interlayer distances (Fig. 5). Specifically, larger adsorbates such as *o*-xylene, *m*-xylene and *p*-xylene sustained higher unit cell volumes through additional adsorptive sites in the intralayer cavities. During layer expansion, shifting of the layers with respect to the positioning of their metal nodes can occur, a mechanism described as layer slippage. While layer slippage often occurs concurrently with layer expansion, it can be pronounced in CNs with bulky pendant groups, such as sql-(azpy)(pdia)-Ni.^96^ Furthermore, interpenetrated 3D structures can have additional modes of movement between individual networks. The most studied interpenetrated families are those of pcu topology, exemplified by pillared layer architectures such as X-pcu-5-Zn, which exhibits 2-fold interpenetration.^91^ During the lp → cp phase transformation the individual networks shift relative to each other to enable open pores in its lp phase and negligible space in its cp phase (Fig. 4c). The phase transformation is driven by noncovalent interactions, as the two individual networks interact through displaced π–π interactions in the cp phase, which is not possible in the lp phase due to the ligands being too far apart (Fig. 4d).

#### Control of flexibility through ligand and metal substitution

Once a parent flexible adsorbent has been identified, crystal engineering can then be employed to systematically fine-tune its composition and, in turn, properties.^97–100^ This is especially so for *P*_go_ and, sometimes, the mechanism of flexibility that dictates the shape of the isotherm. In this context, we have explored linker ligand substitution, which involves replacing the linker with one of comparable size, shape and length. The conformational freedom of the linker was utilised to modulate the position of *P*_go_ for a variety of gases (CO_2_, C_2_H_2_ and C_2_H_4_ at 195 K) in X-pcu-5-Zn. The linker having the highest ability to contort (–CH_2_–CH_2_– spacer) favoured a lower *P*_go_, whereas that with the least conformational freedom (–N=N–) exhibited the highest *P*_go_.^101^ Substitutions of C atoms in phenyl rings with N atoms, *e.g.*X-dia-4-Co and X-dia-5-Co;^83^X-ddi-1-Ni and X-ddi-2-Ni;^82^SIFSIX-23-Cu and SIFSIX-23-Cu^N^,^102^ typically result in lower *P*_go_, and in one case a shape-memory effect was observed.^102^

We have also employed metal substitution as a strategy to tune *P*_go_. In a series of transiently porous networks, X-dmp-1-M (M = Co, Zn, Cd), we found that metal substitution affected the geometries during phase transformations: distorted square pyramidal (in lp) to tetrahedral geometry (in cp) for Co, and retention of tetrahedral and octahedral geometries for Zn and Cd, respectively.^103^ Out of the three analogues studied, Co achieved the lowest *P*_go_ values and Cd the highest (for CO_2_ at 195 K and CH_4_ at 298 K). Even in platforms where MBB distortion is not the main mechanism of flexibility, *e.g.*sql-1-M-NCS (M = Fe, Co, Ni), we have observed that, besides layer expansion, the geometry of pores can change in shape from a rhombus to a square upon CO_2_ sorption, accompanied by coordination sphere distortion.^104^ While all three members exhibited a clear isotherm step, metal substitution affected its position: Fe had the highest *P*_go_, while Ni showed the lowest, indicating that octahedral distortion was more facile for Ni.

#### Next steps in advancing flexible adsorbents

Crystal engineering during the last decade has been used not only to tune and/or improve the properties of existing flexible adsorbent platforms, but also to systematically explore and develop new flexible adsorbents. Therefore, we are now in position to assert the following:

**Truth 1: *“Certain classes of flexible adsorbents are inherently modular and can have their sorption performance parameters controlled by fine-tuning of composition, while design of Gen 1 flexible adsorbents is now possible from first principles.”***

Over the past two decades, flexible adsorbents have progressed from serendipity to systematic design, leading to understanding of structure–function relationships. During the early 2000s, the identification of key components enabling flexibility, such as ligands with contortional flexibility or the ability to isomerise, alongside RBBs and MBBs capable of “butterfly” motions, marked significant developments. These findings allowed for the classification of parameters that can induce flexibility, equipping crystal engineers with the ability to design families of flexible adsorbents from first principles. As a result, the 2010s saw the development of new families of flexible adsorbents where the focus was their properties, particularly in relevance to natural gas storage, water harvesting, and most recently, hydrocarbon separations. In the 2020s, now that we have identified features of flexible adsorbents that can result in promising properties, we have insight for design of the next generation (Gen 3). Concurrently, we can recognise and then address limitations that may hinder performance. One of the key areas requiring further exploration is our inability to accurately predict *P*_go_ for a given combination of flexible adsorbent components. However, once a flexible adsorbent platform is established, *P*_go_ can be systematically tuned to suit a specific application. This tunability is an inherent feature of most flexible adsorbents, and could make them highly adaptable for a broad spectrum of industrial applications as explained below.

## Flexible adsorbents for energy applications

### Natural gas storage

Burning of fossil fuels to produce energy generates over 35 billion tons of carbon dioxide (CO_2_) annually and CO_2_ is the most persistent greenhouse gas.^105,106^ Whereas there is a trend for fossil fuels to produce a smaller share of global energy production in relative terms, increasing global energy production means that in absolute terms the use of fossil fuels continues to grow along with CO_2_ emissions.^107^ Methane (CH_4_), the main component of natural gas (NG), is an attractive intermediate fuel that could facilitate a move towards energy sustainability because it is both more abundant and cleaner as an energy source compared to petroleum and coal. Specifically, CH_4_ produces 40% less CO_2_ emissions than conventional fossil fuels upon burning.^108^ CH_4_ is also attracting attention as a vehicular fuel, not only because of its low carbon emissions, but also thanks to its high octane number (107).^109,110^

The primary challenge for the use of gaseous fuels, such as CH_4_, for transportation lies in their low volumetric density under ambient conditions of temperature and pressure. In particular, at room temperature and 1 atm, CH_4_ has a density of 0.668 kg m^−3^, about 1000 times lower than petroleum (820–870 kg m^−3^).^111^ In order to increase storage density, compressed NG (CNG), liquefied NG (LNG) and adsorbed NG (ANG) are available but there are drawbacks to each. In order to increase the storage density of NG to 430–470 kg m^−3^, LNG requires low temperature (−161.5 °C), while CNG means compression at high pressure (200–250 bar) to achieve storage densities of 160–200 kg m^−3^.^109^ Both CNG and LNG are therefore hindered by cost, energy footprint, space and safety issues. In principle, ANG, which exploits adsorbents to store and deliver CH_4_ at ambient temperatures and lower pressures than CNG (for delivery at 5–35 or 5–65 bar), is advantageous. The CH_4_ storage targets set by the US Department of Energy (DOE) involve gravimetric values of 700 cm^3^ (STP) g^−1^ or 0.5 g g^−1^, or a volumetric target of 350 cm^3^ (STP) cm^−3^.^112^ When omitting the incorporated calculated packing loss of 25%, the volumetric target is 263 cm^3^ (STP) cm^−3^. The problem is that no adsorbent has yet achieved this performance, and a modelling study indicated that that rigid physisorbents are unlikely to exceed volumetric capacity of 200 cm^3^ (STP) cm^−3^.^113^

With respect to the study of CNs in the context of CH_4_ storage, in 1997 Kitagawa's group provided the first report of high-pressure CH_4_ sorption involving a rigid adsorbent, Co_2_(4,4-bipyridine)_3_(NO_3_)_4_,^35^ and followed this up with a study on CuSiF_6_(4,4′-bipyridine)_2_.^114^ Subsequent work tended to focus upon rigid adsorbents with high gravimetric surface areas, including the archetypal MOF HKUST-1(Cu).^115^ The study of flexible adsorbents for NG storage did not occur until 2009 with the study of CH_4_ sorption by MIL-53(Fe)^116^ and gained strong momentum in 2015 with Long's report on the performance of Co(bdp) and Fe(bdp) for CH_4_ storage.^62^ In terms of performance, deliverable capacity, also referred to as working capacity, is a key parameter. High performing adsorbents should minimise gas uptake at deliverable pressure (5 bar), while offering relatively high uptake at the single or dual compressor operable pressures of 35 and 65 bar, respectively. Rigid adsorbents tend not to be ideally suited for CH_4_ storage as their type I isotherms result in retention of gas at low pressure. Flexible adsorbents exhibiting type F-IV isotherms can have isotherm profiles that reduce or eliminate this parasitic CH_4_ uptake at 5 bar. Unfortunately, with few exceptions,^48,62^ transformations from nonporous to porous phases that enable type F-IV isotherm profiles tend to not be extreme enough to afford volumetric working capacities close to 200 cm^3^ cm^−3^, or exhibit phase switching within the needed pressure range. Additionally, to meet the goal of working capacity, the desorption profile needs to be stepped with minimal hysteresis. This section will thus address flexible adsorbents in relation to the following myth:

**Myth 2: *“The nature of flexible adsorbents means that working capacity is unlikely to exceed 200 cm*^*3*^*cm*^*−3*^*.”***

**Fig. 6:**
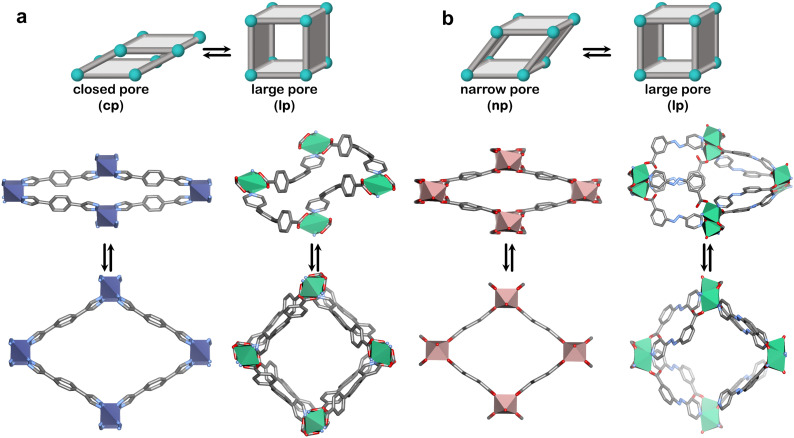
Flexible adsorbents reported for CH_4_ storage and respective phase transformations (a) closed pore (cp) to large pore (lp) for Co(bdp) (left) and X-dia-1-Ni (right) and (b) narrow pore (np) to large pore (lp) for MIL-53(Al)* (left) and X-dia-6-Ni (right). *The reported stepped CH_4_ isotherms correspond to the functionalised **MIL-53(Al)** variant. Crystal structures generated from .cif files archived in the Cambridge Structural Database. CCDC numbers: 1426847, 1426850, 1058444, 1058445, 797263, 797261, 2225286 and 2225290. Atom colour: C, grey; N, blue; O, red; Co, indigo; Ni, green; Al, pink. Hydrogen atoms are omitted for clarity.

**Fig. 7:**
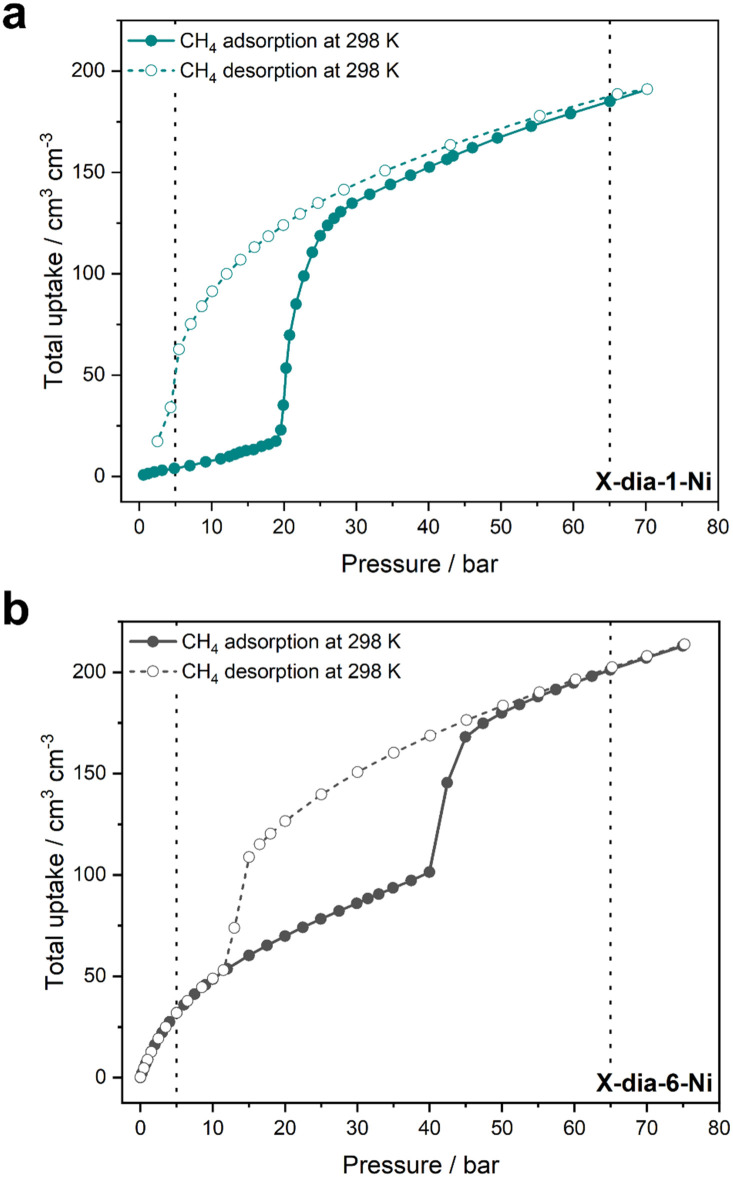
Experimental CH_4_ adsorption and desorption isotherms at 298 K for (a) X-dia-1-Ni and (b) X-dia-6-Ni. Adsorption = full circles, desorption = empty circles. Dashed vertical lines show the range of 5–65 bar. Isotherms were generated from raw data collected in our laboratory.^48,94^

Our pursuit of CH_4_ storage with flexible adsorbents began in 2018 with X-dia-1-Ni.^48^ The X ligand serves two purposes: enabling flexibility, as previously discussed, and large pores in the lp phase to enhance potential CH_4_ uptake. Indeed, we found that X-dia-1-Ni exhibited a type F-IV isotherm through a cp → lp transformation (Fig. 6a and 7a), with CH_4_ uptake of 222 cm^3^ g^−1^ (189 cm^3^ cm^−3^) at 65 bar.^48^ However, the working capacity between 5–65 bar was only 149 cm^3^ cm^−3^ (110 cm^3^ cm^−3^ for 5–35 bar) due to the hysteresis in the desorption branch of the isotherm, resulting in retention of more than 50 cm^3^ cm^−3^ at 5 bar. To regulate this, we prepared mixed crystals through Co substitution, affording a series of mixed crystals of X-dia-1-Ni.^117^ The best performing adsorbent, X-dia-1-Ni_0.89_Co_0.11_, exhibited an uptake of 221 cm^3^ g^−1^ at 65 bar, while also achieving a higher *P*_gc_ than the parent X-dia-1-Ni. As a result, the amount of gas retained at 5 bar was significantly reduced, yielding working capacities of 159 cm^3^ g^−1^ (5–35 bar) and 202 cm^3^ g^−1^ (5–65 bar). Although this was a significant improvement, the previously studied Co(bdp) achieves higher working capacities of 155 cm^3^ cm^−3^ for 5–35 bar and 197 cm^3^ cm^−3^ for 5–65 bar through a cp → lp transformation (Fig. 6a).^62^ The dearth of adsorbents that exhibit extreme cp → lp phase transformations in the relevant pressure range prompted us to explore a flexible adsorbent that exhibits np → lp transformation, X-dia-6-Ni (Fig. 6b).^94^ As anticipated, CH_4_ sorption revealed a type F-II isotherm with an uptake of 235 cm^3^ g^−1^ (200 cm^3^ cm^−3^) at 65 bar (Fig. 7b). While the achieved working capacity (5–65 bar) was only 166 cm^3^ cm^−3^, this study serves as a proof-of-concept for the potential utility of flexible adsorbents with np → lp transformations, which remain understudied. Likewise, a MIL-53 variant, MIL-53(Al)-OH, exhibited a working capacity of 156 cm^3^ g^−1^ from 5–65 bar (np → lp).^118^

Overall, there are now examples of flexible adsorbents that outperform rigid adsorbents in terms of CH_4_ working capacity. This is because, whereas rigid adsorbents such as Ni-MOF-74, HKUST-1**(Cu)**, and UTSA-76a**(Cu)** have higher uptakes at 35 bar than flexible adsorbents, they retain at least a third of their uptakes at 5 bar, reducing their 5–35 bar working capacities to *ca.* 150 cm^3^ cm^−3^.^115,119^ Nevertheless, flexible adsorbents do not yet achieve the DOE targets (197 cm^3^ cm^−3^ for Co(bdp)*vs.* 263 cm^3^ cm^−3^ target). The potential upper limits for CH_4_ working capacities in rigid adsorbents were projected almost a decade ago, with the conclusion that rigid adsorbents are unlikely to meet the targets.^113^ Nevertheless, recent studies suggest that flexible adsorbents offer potential.^120^ The current limitation is their inability to achieve the fully open lp phase when exposed to CH_4_. For example, Co(bdp) exhibits uptake of 672 cm^3^ g^−1^ for N_2_ at 77 K after five gate-opening steps, but only achieves 256 cm^3^ g^−1^ for CH_4_ at 298 K after one gate-opening step.^121^ This limitation might be addressed by reduction of *P*_go_ through crystal engineering, as detailed above.

In summary, although flexible adsorbents are not yet ready to address ANG, rigid adsorbents are inherently disadvantaged due to their isotherm profiles. Moving forward, screening of flexible adsorbents that can sustain *P*_go_ to high porosity lp phases is crucial to meet applicable working capacities. This section suggests the following:

**Truth 2: *“Current flexible adsorbents do not address DOE targets; however, it is unlikely that we have reached the potential upper limit of their working capacity.”***

### Water harvesting

Water and water vapour are ubiquitous and perhaps the most important resources on earth. Water scarcity, from both a quantity and quality perspective, represents an urgent global challenge that is exacerbated by climate change.^122,123^ Harvesting water vapour from air (atmospheric water harvesting, AWH) is an attractive way to tackle water scarcity as the atmosphere contains 12.9 × 10^15^ tons of water^124^ that is inherently replenishable. This is perhaps the most urgent of all global challenges, as water is required to sustain life and agriculture. In addition, water is relevant in other applications including indoor humidity control (dehumidification),^125,126^ food preservation and heating/cooling.^127–129^

Whereas AWH technologies such as fog water collection and dehumidification/condensation are feasible, they are ineffective under dry (low relative humidity, RH) conditions where water is scarce. Traditional desiccants such as zeolites, silica, metal chlorides and metal oxides come with drawbacks: zeolites require high regeneration temperatures due to their hydrophilicity;^130^ silica has generally low uptake due to low surface area;^131^ metal chlorides and oxides require chemical reactions and are prone to deliquescence.^132^ There is therefore an urgent need for the development of improved and energy-efficient AWH technologies.

Regeneration optimised adsorbents (ROSs), *i.e.* adsorbents that offer optimal performance (fast kinetics, low regeneration energy, recyclability, high selectivity, low hysteresis), have emerged in the past decade.^133^ In the context of AWH, water adsorbents have been developed to target two primary applications: (i) AWH at low humidity (5–35% RH); (ii) dehumidification of high humidity air to 40–60% RH. The prototypal high surface area rigid adsorbent HKUST-1(Cu) and a prototypal flexible adsorbent MIL-53(Al) were studied with respect to their water vapour sorption properties in 2002 and 2010, respectively.^134,135^ Whereas HKUST-1(Cu) would be unsuitable for AWH at low RH, MIL-53(Cr) exhibited a stepped isotherm (type F-IV) at low RH, suggesting potential AWH utility for flexible MOFs. Subsequently, a series of rigid MOFs exemplified by Al-fumarate,^136^CAU-10,^137,138^MOF-303^139^ and ROS-037^140,141^ were found to exhibit stepped isotherms at relatively low RH from pore filling and have attracted attention for further development as ROSs. The perception that flexible adsorbents would be unable to be classified as ROSs has perhaps discouraged their study in the context of AWH. Therefore, this section addresses the following myth:

**Myth 3: *“Flexible adsorbents will be unsuitable as ROSs for water harvesting as they are likely to suffer from poor recyclability (mechanical stress), slow kinetics and high hysteresis.”***

**Fig. 8:**
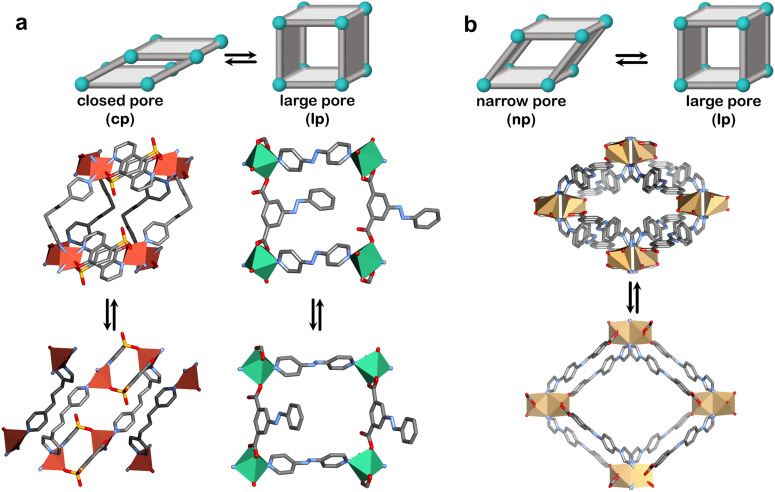
Flexible adsorbents reported for water harvesting and respective phase transformations (a) closed pore (cp) to large pore (lp) for Cu(HQS)(TMBP) (left) and sql-(azpy)(pdia)-Ni (right) and (b) narrow pore (np) to large pore (lp) for X-dia-2-Cd. Crystal structures generated from .cif files archived in the Cambridge Structural Database. CCDC numbers: 2178497, 2212543, 2240522, 2240524. Atom colours: C, grey; N, blue; O, red; S, yellow; Cu, orange; Ni, green; Cd, gold. Hydrogen atoms are omitted for clarity.

**Fig. 9:**
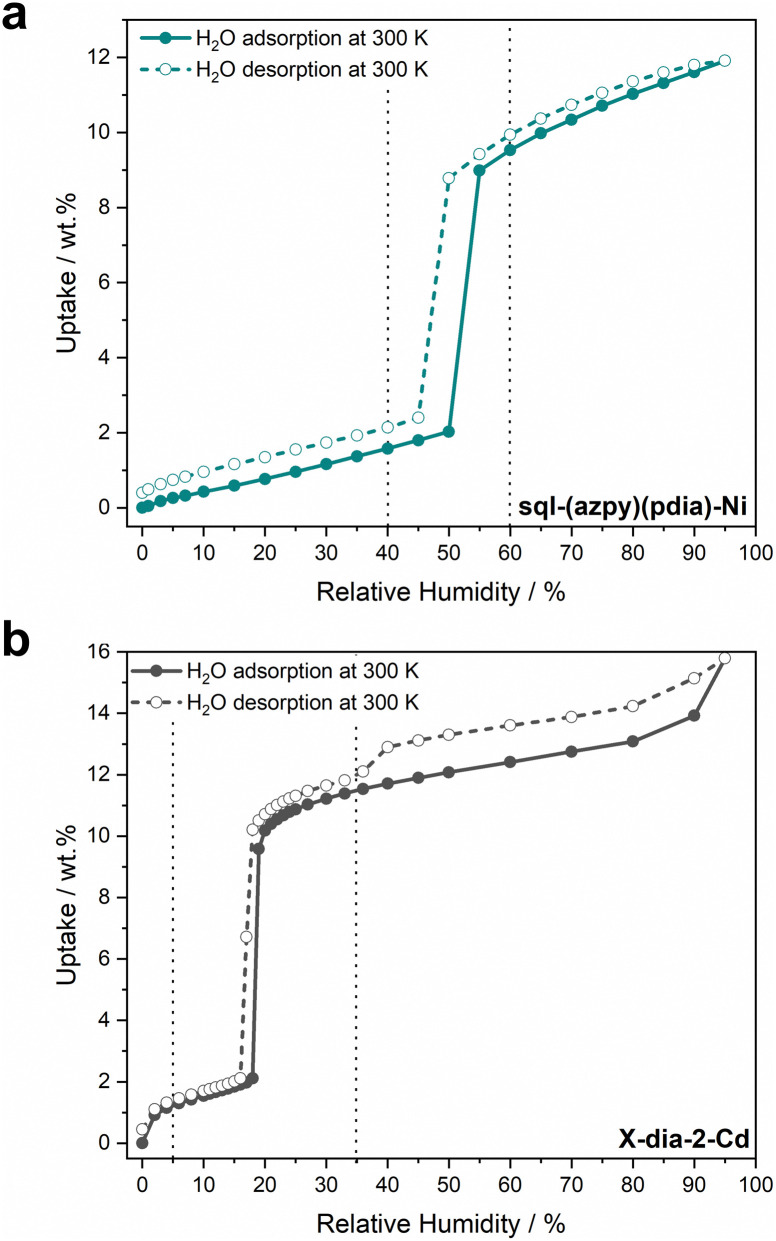
Experimental water adsorption and desorption isotherms at 300 K for (a) sql-(azpy)(pdia)-Ni and (b) X-dia-2-Cd. Adsorption = full circles, desorption = empty circles. Dashed vertical lines show the range of 40–60% RH in (a) and 5–35% RH in (b). Isotherms were generated from raw data collected in our laboratory.^67,96^

To explore water harvesting ROSs, we focused on identifying potentially flexible adsorbents that crystallise in lp phases with 1D channels filled with water. In 2022, we reported a 1D flexible adsorbent, Cu(HQS)(TMBP), synthesised as a hydrated lp phase, which underwent a facile lp → cp transformation upon activation (Fig. 8a).^142^ Water sorption studies revealed a stepped isotherm, with an inflection point at 10% RH, which is relevant for AWH at low humidity, achieving an uptake of 150 mg g^−1^ at 90% RH. The kinetics of loading were also investigated, which revealed comparable kinetics to leading rigid adsorbents such as MOF-303(Al) and CAU-10-H, indicating that concomitant phase transformation did not slow kinetics. A more extensive range of flexible adsorbents were exchanged in water and, in cases where the lp phases could form 1D channels filled with water, water sorption properties were investigated. This led us to the discovery of sql-(azpy)(pdia)-Ni (Fig. 8a), which exhibited a stepped isotherm with a step at 50% RH, identifying it as suitable for indoor humidity control (Fig. 9a).^96^ However, the final uptake was lower than Cu(HQS)(TMBP) (115 mg g^−1^). Most recently, we reported a new flexible adsorbent through similar screening, X-dia-2-Cd (Fig. 8b), which undergoes a np → lp transformation upon water uptake and exhibits a step at 18% RH.^67^ This study prompted us to address the misconceptions regarding flexible adsorbents in the context of water harvesting. We found that X-dia-2-Cd meets the main criteria for potential utility: a stepped isotherm with a step below 30% RH; negligible hysteresis (<5 RH%); mild regeneration temperature (333 K); hydrolytic stability during temperature-humidity swing cycles for 100+ cycles. The saturation uptake of X-dia-2-Cd reached 130 mg g^−1^ at 95% RH (Fig. 9b). Cycling studies on these flexible desiccants demonstrated that they are stable to repeated adsorption–desorption stress: X-dia-2-Cd (128 cycles);^67^ [Cu(HQS)(TMBP)] (>100 cycles);^142^sql-(azpy)(pdia)-Ni (100 cycles).^96^

Compared to rigid adsorbents, the flexible adsorbents studied in our lab are not leading in terms of uptake. For example, the rigid adsorbents MOF-808(Zr)-Br and MOF-303(Al) also have steps below 30% RH but with much higher uptakes: 700 mg g^−1^ and 447 mg g^−1^, respectively.^139,143^ However, kinetics of adsorption/desorption are also key performance indicators.^144^ Recently, our group provided a method for projecting productivity, *i.e.* litres of water per kg of adsorbent per day (L kg^−1^ day^−1^), through heatmaps based on non-equilibrium cycling.^133^ In this context, some rigid adsorbents with high uptake were found to exhibit relatively slow adsorption kinetics, and, consequently, lower productivities than denser adsorbents with lower gravimetric uptake but faster kinetics. For example, compared to MOF-303(Al) (447 mg g^−1^), the rigid adsorbent ROS-039(Zn) achieved about half of the uptake (248 mg g^−1^), but exhibited faster kinetics. That loading was found to be controlled by surface diffusion to the adsorbent bed means that adsorbents with a step at lower % RH will adsorb at a faster rate. Through non-equilibrium cycling, productivities of 1.3 and 7.3 L kg^−1^ d^−1^ were found for MOF-303(Al) and ROS-039(Zn), respectively. Given that kinetics of water sorption, as well as non-equilibrium cycling, has rarely been studied in flexible adsorbents, they cannot yet be benchmarked as AWH adsorbents.

In summary, whereas rigid adsorbents can achieve higher water vapour uptake than flexible variants, uptake is only part of the picture. Considering that the study of flexible adsorbents as desiccants has only recently attracted attention, it is too early to assert if flexible adsorbents can be suited for AWH. This section supports the following assertion:

**Truth 3: *“Flexible adsorbents remain understudied with respect to AWH applications and it is too early to judge how well they compete with rigid adsorbents in terms of performance.”***

### Separations

Purification of speciality and commodity chemicals is an energy-intensive step in their manufacture because conventional processes that involve distillation, solvent extraction or catalytic conversion are inherently energy intensive and wasteful in terms of by-products.^145^ Adsorbents, especially physisorbents, offer an opportunity to greatly reduce the energy and environmental footprint of chemical purification processes. This is because separation by physisorbents is based on the molecular properties of the gas mixture components, rendering adsorptive separation up to 10 times more energy-efficient than traditional methods.^109^

However, adsorptive separation is not as easy as it sounds. This is because most porous physisorbents tend not to have high enough selectivity to address removal of trace impurities. This is because “*breaking up is hard to do*”,^146^ especially for (i) molecules with similar physicochemical properties and (ii) trace impurities for which high selectivity is a necessity. For example, polymer-grade ethylene (C_2_H_4_) contains acetylene (C_2_H_2_) as a trace impurity (*ca.* 1%) that must be reduced to levels below 5 ppm.^147^ Similarly, as-produced propylene (C_3_H_6_) contains trace (*ca.* 1%) propyne (C_3_H_4_), which must be reduced below 5 ppm prior to catalytic polymerisation to polypropylene.^148^ Therefore, a suitable physisorbent must not only be able to distinguish between components with similar physicochemical properties, they must be effective at trace concentrations of the impurity. The challenge of separations is apparent from the kinetic diameters of common chemical commodities that must be purified: CO_2_, 3.3 Å; N_2_, 3.6 Å; CH_4_, 3.8 Å; C_2_H_2_, 3.3 Å; C_2_H_4_, 4.2 Å; C_2_H_6_, 4.4 Å; C_3_H_8_, 4.3 Å; C_3_H_6_, 4.7 Å; C_3_H_4_, 4.8 Å; *p*-xylene, 5.8 Å; ethylbenzene, 5.8 Å; *o*-xylene, 6.8 Å; *m*-xylene, 6.8 Å.^149^

**Fig. 10:**
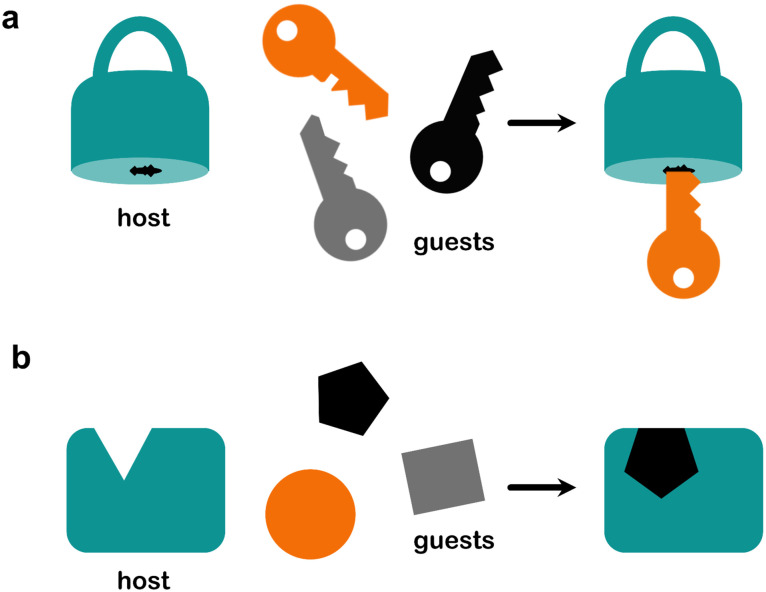
(a) Lock and key binding for a rigid adsorbent and (b) induced fit binding for a flexible adsorbent.

Rigid physisorbents with ultra-high selectivity have emerged over the past decade and resulted in dramatic improvements in performance for several commodity separation processes: CO_2_/N_2_; CO_2_/CH_4_; C_2_H_2_/CO_2_; C_2_H_2_/C_2_H_4_; C_2_H_6_/C_2_H_4_; C_3_H_4_/C_3_H_6_; C8 isomers.^150–152^ In particular, the 2010s saw HUMs that could effect “*gas separations at will*”.^153^ HUMs, comprised of inorganic and organic linkers, have outperformed previous benchmark adsorbents, sometimes by one or more orders of magnitude in terms of selectivity. For example, the first adsorbent reported to selectively adsorb C_2_H_2_ from a C_2_H_2_/CO_2_ mixture was reported by Kitagawa's group in 2005, namely Cu_2_(pzdc)_2_(pyz).^154^SIFSIX-3-Zn reported in 2013 by our group^39^ improved selectivity for CO_2_/N_2_ by one order of magnitude compared to previous benchmarks such as UTSA-16**(Co)**,^155^ and Mg-MOF-74 (Mg/DOBDC or CPO-27-Mg).^156,157^ HUMs are now the benchmarks for a variety of hydrocarbon separations, and generally function though the presence of a high density of bespoke identical binding sites.^30^ In effect, these rp adsorbents have the right pore size, shape and chemistry for a specific separation.^158^ The binding site interactions can be described as being analogous to the “lock and key” mechanism from biochemistry,^159,160^*i.e.* the adsorbate binds to the adsorbent in a manner similar to tight substrate binding by enzymes, where ultra-high selectivity is also needed (Fig. 10a).

Nevertheless, there are limitations to rigid adsorbents. First, real world gas mixtures are non-binary, and so performance can be affected by the other components, especially water vapour.^161–163^ Second, higher density adsorbents tend to have relatively low gravimetric uptakes and so a trade-off tends to exist between uptake, selectivity and binding affinity.^164^ In principle, flexible adsorbents can address this trade-off and combine high uptake with high selectivity, especially if they can engage in induced fit binding of adsorbates (Fig. 10b), *i.e.* where the adsorbent adapts to the shape and size of the adsorbate in order to facilitate optimal binding.^165,166^ This section addresses recent progress concerning the potential utility of flexible adsorbents in separations by addressing the following myth:

**Myth 4: *“Flexible adsorbents will have poor selectivity once they transform to alpphase.”***

**Fig. 11:**
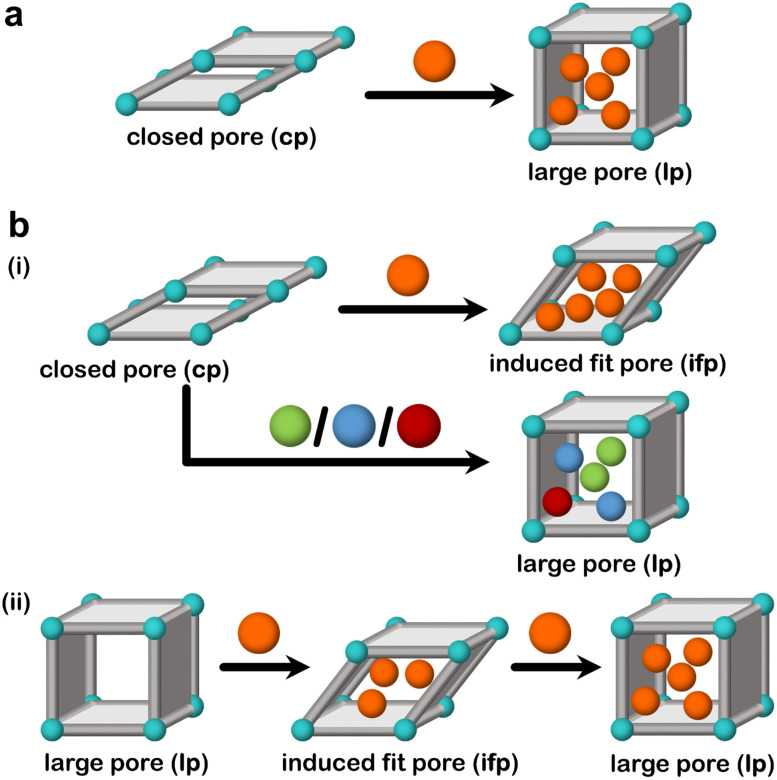
Schematic representations for (a) switching adsorbents that undergo closed pore (cp) to large pore (lp) phase transformation and (b) induced fit adsorbents, (i) induced fit adsorbent that undergoes closed pore (cp) to induced fit pore (ifp) phase transformation for one adsorbate out of a mixture and (ii) induced fit adsorbent that shows shrinkage from large pore (lp) to induced fit pore (ifp) phase towards an adsorbate.

To date, only a few flexible adsorbents are known to exhibit induced fit behaviour.^59,65,68,167^ Induced fit binding is a feature of some flexible adsorbents and differs from the more commonly observed guest-induced cp → lp or np → lp transformations (Fig. 11a). Generally, we can classify induced fit adsorbents into two categories:

(a) Category I: adsorbents that undergo a bespoke structural transformation for one guest out of a mixture, leading to an induced fit phase that exists only for that particular guest;

(b) Category II: adsorbents that undergo a bespoke structural transformation for one guest out of a mixture, leading to an induced fit phase that would not correspond to the energy minimum in the absence of that guest.

Category I adsorbents are exemplified by those that undergo induced fit mechanisms towards a particular guest leading to an induced fit pore (ifp), but undergo phase transformation to the as-synthesised lp phase for other guests under the same conditions (Fig. 11b(i)). In category II adsorbents, the guest enforces a shrinkage on the adsorbent, which is not the energetically favoured phase in the absence of the guest (Fig. 11b(ii)). This leads to a lp → np → lp transformation, and, when this is true for only one particular guest, then the np can be viewed as an induced fit phase (ifp), classifying the transformation as lp → ifp → lp. Examples of such adsorbents will be discussed below.

### C_2_H_2_/CO_2_ and C_2_H_6_/C_2_H_4_ separation

**Fig. 12:**
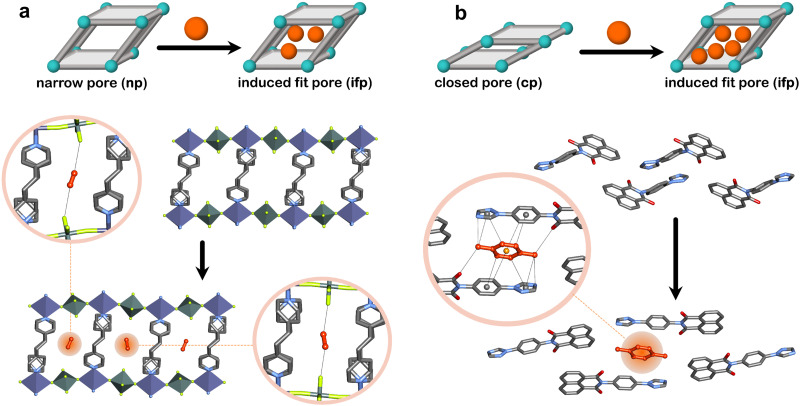
Flexible adsorbents reported for hydrocarbon separations showing induced fit binding: (a) narrow pore (np) to induced fit pore (ifp) phase transformation in sql-SIFSIX-bpe-Zn triggered by acetylene and (b) closed pore (cp) to induced fit pore (ifp) phase transformation in TPBD triggered by *p*-xylene. Crystal structures generated from .cif files archived in the Cambridge Structural Database. CCDC numbers: 2088146, 2048415, 2243659 and 2243662. Atom colours: C, grey; N, blue; O, red; S, yellow; F, neon green; Si, purplish grey; Zn, purple. Guest molecules are shown in ball and stick representations (C, orange). Hydrogen atoms are omitted for clarity.

Separation of C_2_H_2_ and CO_2_ is particularly challenging due to their similar molecular size (*ca.* 3.3 Å) and boiling points (189 and 195 K respectively). Therefore, the design of adsorbents for this purpose should focus on host–guest interactions for preferential binding rather than size exclusion. Our contributions initially focused on the development of HUMs that can serve as rigid adsorbents with high C_2_H_2_/CO_2_ selectivity at 100 kPa and 298 K: NKMOF-1-Ni (23.9),^168^DICRO-4-Ni-i (13.9),^169^SIFSIX-21-Cu (10.0)^164^ and NbOFFIVE-3-Cu (9.5).^164^ Recently, we used a flexible ligand to develop a flexible adsorbent that combines the enhanced electrostatics of HUMs with the flexibility of conventional flexible adsorbents.^65^ We found that sql-SIFSIX-bpe-Zn exhibited induced fit binding for C_2_H_2_, the np phase slightly contracting to fit guest molecules into an ifp phase (Fig. 12a). Upon full loading, the network transformed to the lp phase. Exposure to CO_2_ under the same conditions resulted in np → lp transformation as is typical for flexible adsorbents. The mechanism of binding was supported by contortion of the linker, as well as host–guest interactions (hydrogen bonding, Fig. 12a). In terms of separation, the induced fit binding resulted in record-high heat of adsorption (*Q*_st_) for C_2_H_2_ of 67.5 kJ mol^−1^, while C_2_H_2_/CO_2_ selectivity reached 8.4, enabling sql-SIFSIX-bpe-Zn to separate an equimolar binary mixture.

In order to produce polymer grade C_2_H_4_, C_2_H_6_ impurities must also be removed. Most adsorbents reported to date are C_2_H_4_/C_2_H_6_ selective due to the higher quadrupole moment of C_2_H_4_ and the presence of π electrons. However, preferential adsorption of C_2_H_4_ implies the need for additional desorption steps to obtain C_2_H_4_ as an effluent. A potential solution to this problem is the design of adsorbents which are “inverse selective” for C_2_H_6_. This concept was introduced in 2010 by a seminal study on the flexible adsorbent ZIF-7, which demonstrated “inverse selectivity” for a C_2_H_6_/C_2_H_4_ mixture.^170^ The benchmark inverse selective adsorbents have C_2_H_6_/C_2_H_4_ selectivities of 2–4 at 298 K and 100 kPa: Fe_2_(O_2_)(dobdc) (4.4);^171^Cu(Qc)_2_ (3.4);^172^NPU-3 (3.2);^173^ZJU-120 (2.7; at 296 K);^174^MAF-49 (2.7),^175,176^ZIF-7 (1.5).^170,175,176^ Recently, we discovered that a flexible adsorbent, the mixed crystal X-dia-1-Ni_0.89_Co_0.11_, offers proof-of-principle for flexible adsorbents in this context.^69^ Gas sorption revealed gate-opening towards C_2_H_6_ at 273 K, but no gate-opening towards C_2_H_4_ under the same conditions, corresponding to a cp → lp transformation only for C_2_H_6_. Selective gate-opening towards C_2_H_6_ resulted in corresponding C_2_H_6_/C_2_H_4_ selectivity of 5.5, which was also supported by dynamic column breakthrough experiments. Although the study was not performed at ambient (298 K) temperature, it reiterates that flexible adsorbents can exhibit benchmark molecular recognition properties, in some cases leading to inverse selectivity.

### C8 isomer separations

Purification of the C8 isomers, *o*-xylene (OX), *m*-xylene (MX), *p*-xylene (PX), and ethylbenzene (EB), is necessary since they are typically produced as mixtures. Separation of C8 isomers in our group has generally been investigated through Werner complexes or CNs. Among these are flexible adsorbents that offer selective capture of OX, exemplified by the Werner complex SAMM-3-Cu-OTf (*S*_OX/PX_ = 23.1)^177^ and the CN sql-1-Co-NCS (*S*_OX/PX_ = 9.6).^64^ Although the current benchmark is another Werner complex, SAMM-3-Ni-NCS, otherwise known as [Ni(NCS)_2_(ppp)_4_] (*S*_OX/PX_ = 40.5),^178^ its uptake is lower than SAMM-3-Cu-OTf (420 mg g^−1^) and sql-1-Co-NCS (852 mg g^−1^). Last year, we unexpectedly found that a nonporous organic molecular solid can exhibit induced fit binding towards PX. Specifically, the host–guest complex of TPBD could only be formed with PX through a cp → ifp phase transformation, while OX, MX, and EB, were excluded (Fig. 12b).^68^ The induced fit binding enabled high selectivity over OX (*S*_PX/OX_ = 76.1) which compares to the leading rigid, MAF-89 (*S*_PX/OX_ = 221),^179^ and flexible, Mn-dhbq (*S*_PX/OX_ = 76.9) adsorbents.^180^ Even though MAF-89 leads for *S*_PX/OX_, it was outperformed by TPBD for selectivity towards MX (*S*_PX/MX_ = 22.1 for TPBD). The induced fit binding mechanism also enabled relatively high gravimetric uptake by TPBD (160 mg g^−1^) and recyclable separation of C8 isomers.

The sum of this work suggests that flexible adsorbents not only have the potential for highly efficient hydrocarbon separation, but also that their ability to selectively bind guests through induced fit mechanisms could make flexible adsorbents uniquely suited for trace separations. Therefore, myth 4 can be addressed as follows:

**Truth 4: *“Just as in biochemistry, the induced fit mechanism in flexible adsorbents can result in highly selective binding in a manner that cannot be achieved by lock-and-key binding.”***

## Conclusions and perspectives

While rigid adsorbents have been studied and extensively optimised over the past 25 years, flexible adsorbents have only recently been regarded as serious candidates for utility. This contribution highlights how flexible adsorbents not only compete with traditional rigid adsorbents, but can surpass them if the right adsorbent is selected for the right application. This is because their inherent flexibility is accompanied by properties that cannot be exhibited by rigid adsorbents, such as enhanced working capacity, heat management and potential for induced fit binding. Although several of the concerns about flexible adsorbents persist, some flexible adsorbents are hydrolytically stable, recyclable, and exhibit benchmark selectivity towards specific guests. While some myths are still in play, *e.g.* the potential upper limit of working capacity, others can now be debunked. Notably, design and control principles for flexible adsorbents are now in hand and their properties can make them well-suited as ROSs and/or induced fit binding.

That insights into the mechanisms of flexibility were gained from the discovery phase (Phase 1) has enabled deliberate design or screening of other Gen 1 flexible adsorbents. Although Gen 1 adsorbents are now widely available, their properties, particularly whether *P*_go_ will occur in the desirable pressure range, remain difficult to predict. However, that tuning of *P*_go_ has nowadays become controllable, underscores that significant progress has been achieved over the last decade. We are therefore perhaps now approaching the end of the structure/function phase (Phase 2) which deals with the predictability and reproducibility of properties. Further translation of the properties of some identified candidates is now required to broaden the scope of flexible adsorbents. Herein we see two key areas that require more work: (i) non-equilibrium cycling, which could potentially optimise adsorption kinetics and enhance productivity, has not been thoroughly investigated in flexible adsorbents; (ii) the potential of induced fit binding in flexible adsorbents for separations, which has been introduced only in the last five years, requires further insight given that adsorbents that can adapt to a specific adsorbate could offer superior performance compared to traditional rigid adsorbents.

We assert that we are now at the beginning of the “utility phase” but there remain unresolved challenges. The next major step for flexible adsorbents is not only to make them better, but also cheaper and greener, as these are prerequisites for large-scale applications that might address global challenges. In terms of formulation of flexible adsorbents for practical applications, two areas require attention. First, the effect of particle size, which directly impacts not just adsorbent kinetics, but in some cases whether flexibility can occur in the first place. Second, current methods for screening adsorbents are time-consuming, being reliant on serial sorption experiments or computational simulations that can take weeks or months. It is therefore essential to develop faster, more efficient screening methods to accelerate the discovery and optimisation of flexible adsorbents especially as many existing structures deposited in the CSD may exhibit flexibility and remain to be investigated.

To conclude, a few promising flexible adsorbents with benchmark properties have now been identified, indicating that others with equal or even better performance remain to be discovered. The potential utility of flexible adsorbents could extend well beyond initial interest in ANG, including into societally important areas such as AWH and hydrocarbon separations. We therefore feel that it is time to challenge the misconceptions that surround flexible adsorbents and encourage a paradigm shift in terms of both scientific study and technological utility.

## Data availability

There are no data to support this Feature Article.

## Conflicts of interest

There are no conflicts to declare.
